# Dynamic characteristics and functional analysis provide new insights into long non-coding RNA responsive to *Verticillium dahliae* infection in *Gossypium hirsutum*

**DOI:** 10.1186/s12870-021-02835-8

**Published:** 2021-02-01

**Authors:** Guoning Wang, Xingfen Wang, Yan Zhang, Jun Yang, Zhikun Li, Lizhu Wu, Jinhua Wu, Nan Wu, Lixia Liu, Zhengwen Liu, Man Zhang, Liqiang Wu, Guiyin Zhang, Zhiying Ma

**Affiliations:** State Key Laboratory of North China Crop Improvement and Regulation, Hebei Agricultural University, Baoding, 071001 China

**Keywords:** Cotton, lncRNA, *Verticillium dahliae*, Resistance, LOX

## Abstract

**Background:**

Verticillium wilt is a widespread and destructive disease, which causes serious loss of cotton yield and quality. Long non-coding RNA (lncRNA) is involved in many biological processes, such as plant disease resistance response, through a variety of regulatory mechanisms, but their possible roles in cotton against *Verticillium dahliae* infection remain largely unclear.

**Results:**

Here, we measured the transcriptome of resistant *G. hirsutum* following infection by *V. dahliae* and 4277 differentially expressed lncRNAs (delncRNAs) were identified. Localization and abundance analysis revealed that delncRNAs were biased distribution on chromosomes. We explored the dynamic characteristics of disease resistance related lncRNAs in chromosome distribution, induced expression profiles, biological function, and these lncRNAs were divided into three categories according to their induced expression profiles. For the delncRNAs, 687 *cis*-acting pairs and 14,600 *trans*-acting pairs of lncRNA-mRNA were identified, which indicated that *trans*-acting was the main way of Verticillium wilt resistance-associated lncRNAs regulating target mRNAs in cotton. Analyzing the regulation pattern of delncRNAs revealed that *cis*-acting and *trans*-acting lncRNAs had different ways to influence target genes. Gene Ontology (GO) enrichment analysis revealed that the regulatory function of delncRNAs participated significantly in stimulus response process, kinase activity and plasma membrane components. Kyoto Encyclopedia of Genes and Genomes (KEGG) enrichment analysis indicated that delncRNAs participated in some important disease resistance pathways, such as plant-pathogen interaction, alpha-linolenic acid metabolism and plant hormone signal transduction. Additionally, 21 delncRNAs and 10 target genes were identified as being involved in alpha-linolenic acid metabolism associated with the biosynthesis of jasmonic acid (JA). Subsequently, we found that *GhlncLOX3* might regulate resistance to *V. dahliae* through modulating the expression of *GhLOX3* implicated in JA biosynthesis. Further functional analysis showed that *GhlncLOX3*-silenced seedlings displayed a reduced resistance to *V. dahliae*, with down-regulated expression of *GhLOX3* and decreased content of JA.

**Conclusion:**

This study shows the dynamic characteristics of delncRNAs in multiaspect, and suggests that *GhlncLOX3*-*GhLOX3*-JA network participates in response to *V. dahliae* invasion. Our results provide novel insights for genetic improvement of Verticillium wilt resistance in cotton using lncRNAs.

**Supplementary Information:**

The online version contains supplementary material available at 10.1186/s12870-021-02835-8.

## Background

Cotton is one of the most important economic crops for its natural fiber and oil seed, and has been widely cultivated around the world. The quality and yield of cotton are frequently subjected to the serious threat from Verticillium wilt which is one of the most destructive diseases in cotton and deserves enormous researches to control efficiently [1]. Verticillium wilt is caused by *Verticillium dahliae*, a soil-borne, xylem-invading, hemi-biotrophic fungal pathogen. More than 400 plant species were invaded by *V. dahliae*, causing an intractable vascular wilt disease and even death [1, 2]. Its microsclerotia can survive in soil for 15 years [3, 4], becoming a serious challenge to the control on Verticillium wilt.

Jasmonic acid (JA), as a vital plant hormone, plays an important regulatory role in plant responses to biotic and abiotic stresses. Increasing evidence has shown that the perception of pathogen attack promotes synthesis of JA from lipid, and the accumulation of JA activates downstream defensive genes expression in plant, thereby protects plant against the attacker [5, 6]. Using JA-insensitive mutants, it has been found that JA-dependent defense pathways in Arabidopsis contribute to resistance against the fungal pathogens *Alternaria brassicicola* and *Botrytis cinerea* [7]. It has been reported that JA response can reduce tomato susceptibility to *V. dahliae* and *Fusarium oxysporum* [8]. Importantly, it has been demonstrated that JA response pathway is vital to confer Verticillium wilt resistance in cotton [9–11].

In recent years, high-throughput technologies have been widely used to monitor expression profiles and identify a large number of differentially expressed genes/proteins in cotton inoculated with *V. dahliae*. Based on transcriptional analysis and proteomic analysis, it has been revealed that plant hormone signal transduction, phenylpropanoid biosynthesis pathway, lignin metabolism, cell wall relating enzymes/proteins, adenosine triphosphate (ATP)-binding cassette (ABC) proteins, reactive oxygen species and gossypol play important roles in the fight against *V. dahliae* in cotton [11–18]. In addition, a large number of Verticillium-resistance genes and proteins were identified. For example, previously we obtained 3027 Verticillium-resistance unigenes in *G. barbadense* cv Pima90–53 [13], and identified 1717 and 1476 differentially abundant proteins in resistant *G. hirsutum* cv. ND601 and susceptible *G. hirsutum* cv. CCRI8 after infection with *V. dahliae*, respectively [17]. Moreover, there were some microRNAs (miRNAs) identified related to cotton defense against *V. dahliae* by high-throughput sequencing [19]. These studies not only provide substantial information for research on molecular mechanism of cotton resistance to Verticillium wilt, but also lay a foundation for understanding the function of long non-coding RNA (lncRNA) in regulating plant response to *V. dahliae* infection.

LncRNAs are longer than 200 nucleotides (nt) RNAs without protein-coding ability [20]. LncRNAs play important roles in flowering time regulation [21–24], photomorphogenesis [25], reproductive development [26], fruit development [27–29], biotic and abiotic stress responses [30–32]. Recently, the roles of lncRNAs in plant-pathogen interaction have attracted much attention. In wheat, lncRNAs participate in the response to powdery mildew and stripe rust infection [33, 34]. Several lncRNAs responding to *F. oxysporum* infection have been identified in Arabidopsis [30]. A total of 110 lncRNAs responding to phytoplasma infection have been identified in Paulownia by high-throughput sequencing [35]. It has been found that a number of lncRNAs are involved in response to *Sclerotinia sclerotiorum* infection in *Brassica napus* [36]. LncRNA *ELENA1* has been shown to regulate *PATHOGENESIS-RELATED GENE1* (*PR1*) through interacting with Mediator subunits 19a (MED19a), resulting in enhancing Arabidopsis resistance to *Pseudomonas syringe* [37]. More recently, it has been found that tomato lncRNA23468 can execute function as a competing endogenous RNA to modulate *NBS-LRR* genes by decoying miR482b in the interaction between tomato and *Phytophthora infestans* [38].

As was previously reported, a number of functional lncRNAs were detected in cotton. It was discovered that 1296 lncRNAs were related to fiber initiation, and 720 lncRNAs played a role in fiber elongation and secondary cell wall biosynthesis [39, 40]. Some lncRNAs were identified in *G. hirsutum* as being involved in regulating plant hormone against drought stress [41]. A lncRNA (lncRNA973) was confirmed to play an important role in increasing salt tolerance of cotton [42]. Furthermore, 1236 and 1907 lncRNAs were found in response to *V. dahliae* infection in resistant *G. barbadense* and susceptible *G. hirsutum*, respectively, and lineage-species specific (LS) lncRNAs were identified in response to the disease [1]. However, the function and dynamic characteristics of lncRNAs in resistant *G. hirsutum* remain unknown.

In this study, we used RNA-seq technology to excavate Verticillium-resistance lncRNAs in resistant *G. hirsutum* and revealed the function of important lncRNAs by virus-induced gene silencing (VIGS) technology. We explored the dynamic characteristics of lncRNAs associated with disease response in some aspects, such as chromosome distribution, induced expression profiles, biological function, and linked the infection process by *V. dahliae* to lncRNAs dynamic expression profiles. *Trans*-acting was indicated as the main way of Verticillium wilt resistance-associated lncRNAs regulating the target mRNAs in cotton. The important function of *GhlncLOX3* involved in cotton resistance to *V. dahliae* via *GhlncLOX3*-*GhLOX3*-JA network was revealed.

## Results

### Up to 4277 lncRNAs were induced expression in cotton roots after inoculation with *V. dahliae*

In order to investigate the transcriptome of resistant *G. hirsutum* in response to *V. dahliae* infection, total RNAs isolated from *V. dahliae*-inoculated plants (cv. Nongda601) at 2, 6, 12, 24 and 48 h post inoculation (hpi) (VD2hpi, VD6hpi, VD12hpi, VD24hpi, and VD48hpi) were sequenced using a high-throughput RNA-seq approach. For comparison, the transcriptomes of mock-treated samples (MT2hpi, MT6hpi, MT12hpi, MT24hpi, and MT48hpi) were also sequenced. Through quality filters, about one billion clean reads were obtained. By transcript assembly, differential isoform and gene expression analysis using Cufflinks, approximately 77.1 to 83.3% of these clean reads were aligned to the reference genome of *G. hirsutum* [43]. Approximately 89% of the aligned reads were uniquely mapped to a single genomic locus, attesting to the high quality of the RNA-seq reads and the reference cotton genome (Additional file 1: Table S1). By cufflinks assemblies, 380,559 transcripts were obtained ultimately, of which 16,876 were calculated lncRNAs.

**Fig. 1:**
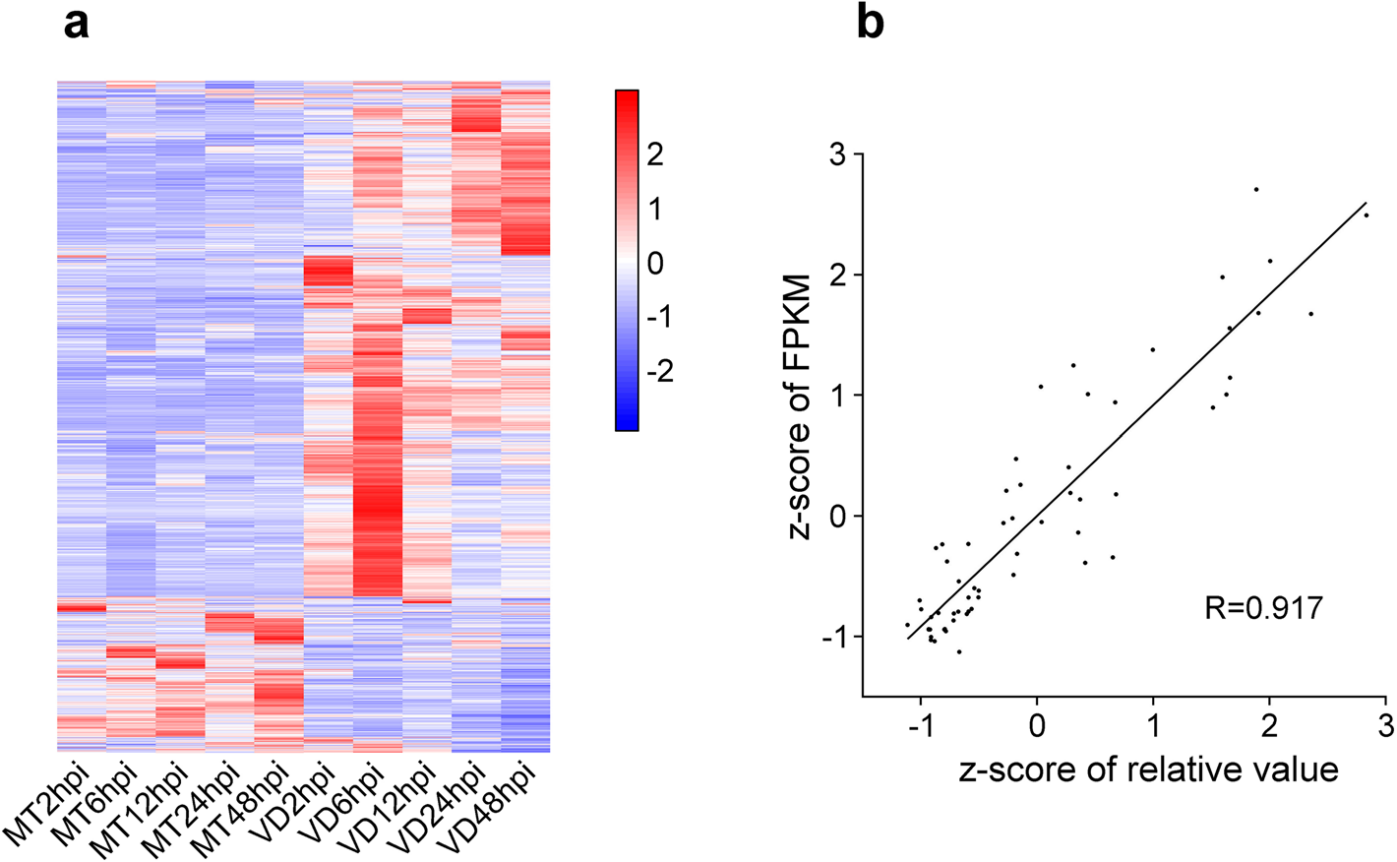
Identification of differentially expressed lncRNAs. **a** Heat map shows the expression profiles of the delncRNAs in all samples. The expression levels of each gene in different samples are normalized. **b** Correlation between qRT-PCR (X-axis) and Fragments Per Kilobase of exon per Million fragments mapped (FPKM) from sequencing data (Y-axis) for six randomly selected lncRNAs

LncRNAs with a greater than 2-fold expression change and *q*-value < 0.05 were recognized as differentially expressed. As a result, 4277 delncRNAs were identified (Fig. 1a). To validate the findings from sequencing data, the expression levels of six delncRNAs, randomly chosen, were assessed using qRT-PCR in all samples. The results from RNA sequencing and qRT-PCR showed a strong correlation (*R* = 0.917, *p* < 0.01) (Fig. 1b), suggesting the high quality of the RNA-seq.

### DelncRNAs showed obvious dynamic characteristics in chromosome distribution and induced expression profiles

**Fig. 2:**
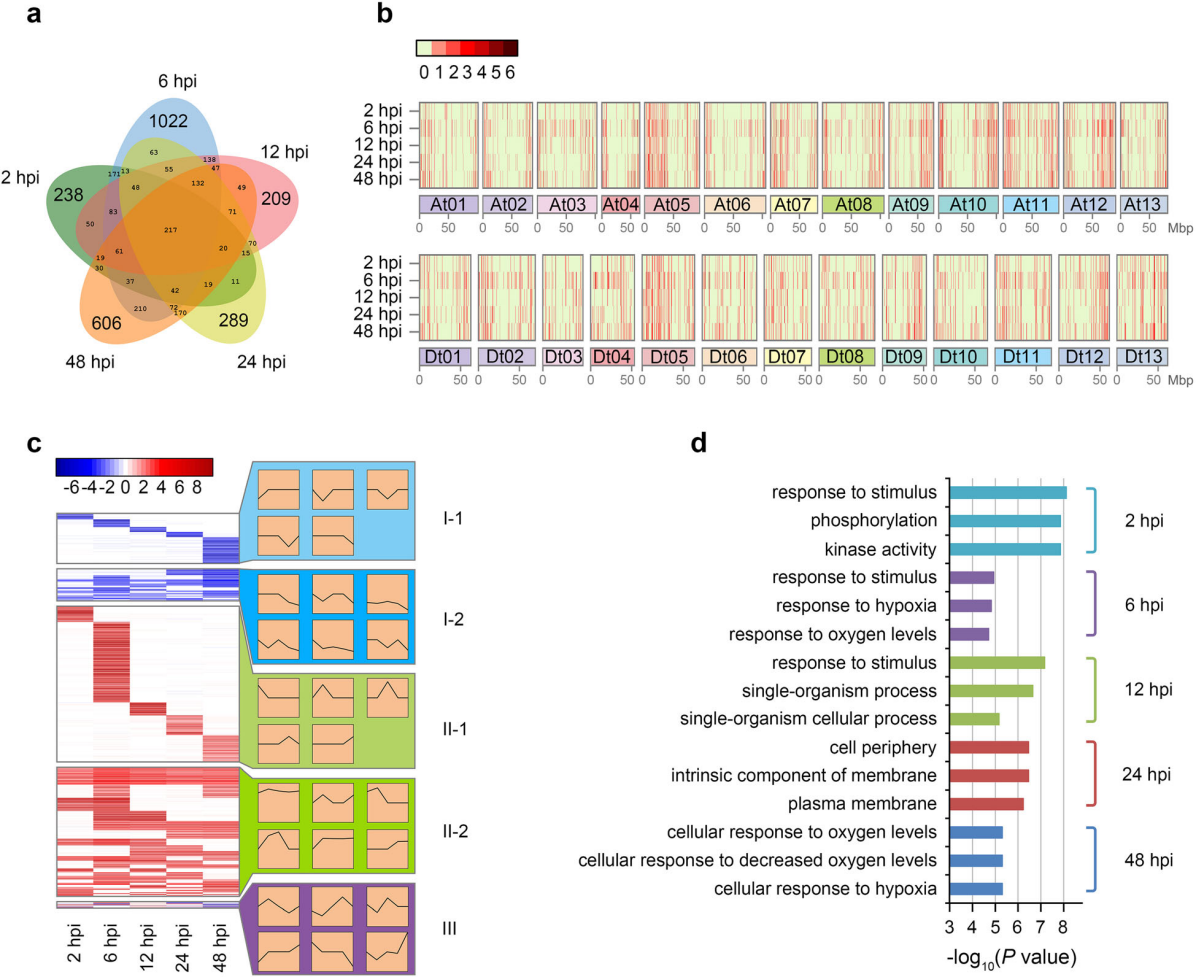
Dynamic characteristics of differentially expressed lncRNAs. **a** The Venn diagram, drawing from 4277 delncRNAs, highlights the unique delncRNAs at five time points. **b** DelncRNAs density and distribution in cotton genome at five time points. The number of delncRNAs per 500-kb is shown as color index. **c** Induced expression profiles (left-half) and patterns (right-half) of delncRNAs after inoculation of *Verticillium dahliae*. Color index depicting the fold up- or down-regulation of lncRNAs induced by *V. dahliae*. Only partial expression patterns are shown here, and the other patterns are shown in Additional file 2: Fig. S1. **d** Gene Ontology (GO) enrichment analysis of unique delncRNAs. Only the top three of the significantly enriched GO terms are shown at each time point. The enrichment method is the same as Fig. 4

Through comparison of the number of delncRNAs at each of the five time points, we found that the abundance of delncRNAs differed evidently. A total of 1074, 2411, 1284, 1307 and 1802 lncRNAs were differentially expressed at 2 hpi, 6 hpi, 12 hpi, 24 hpi and 48 hpi, respectively. And these delncRNAs were mainly up-regulated, especially at 6 hpi, up to 85.9% (Table 1). These delncRNAs exhibited a high degree of temporal specificity: substantial number of delncRNAs were unique to 2 hpi, 6 hpi, 12 hpi, 24 hpi, or 48 hpi. Especially at 6 hpi, about 42.4% of delncRNAs (1022) were unique (Fig. 2a).

**Table 1:** Differentially expressed lncRNAs between *V. dahliae*-inoculated vs mock-treated

delncRNA	2 hpi	6 hpi	12 hpi	24 hpi	48 hpi
Up-regulated	881 (82.0%)	2071 (85.9%)	1072 (83.5%)	1034 (79.1%)	1179 (65.4%)
Down-regulated	193 (18.0%)	340 (14.1%)	212 (16.5%)	273 (20.9%)	623 (34.6%)
Total	1074	2411	1284	1307	1802

Localization analysis revealed that the distribution of lncRNAs induced by *V. dahliae* was not uniform on chromosomes. There were higher densities of delncRNAs on At05, At11 and Dt05, and lower on At06, Dt10 and At02. In addition, the delncRNAs tended to be distributed on chromosome ends. We also found that the distribution of delncRNAs on chromosomes varied with the infection process of *V. dahliae* (Fig. 2b).

All of the 4277 delncRNAs were divided into three categories according to their induced expression profiles, and each category had some distinctively induced expression patterns (Fig. 2c). Type I and type II clusters represented down- and up-regulated lncRNAs induced by *V. dahliae*, approximately 21.7 and 76.7%, respectively. Even though only 68 chimeric-regulated lncRNAs were classified as type III, they had the most complex expression patterns upon *V. dahliae* infection. For instance, there were 10 lncRNAs which were up-regulated at 6 hpi and then down-regulated at later one or two time points. Type I and type II clusters could be divided into two subclasses, respectively. We found that delncRNAs in the subtypes I-1 and II-1 clusters were down-regulated and up-regulated at only one time point, respectively. While delncRNAs in subtypes I-2 and II-2 clusters, more complicated, were down-regulated and up-regulated at least two time points and the induction rate varied with the infection times. These results suggested that delncRNAs had obvious dynamic characteristics in cotton after inoculation with *V. dahliae*.

### *Trans*-acting was the main way of delncRNAs regulating the target mRNAs

**Fig. 3:**
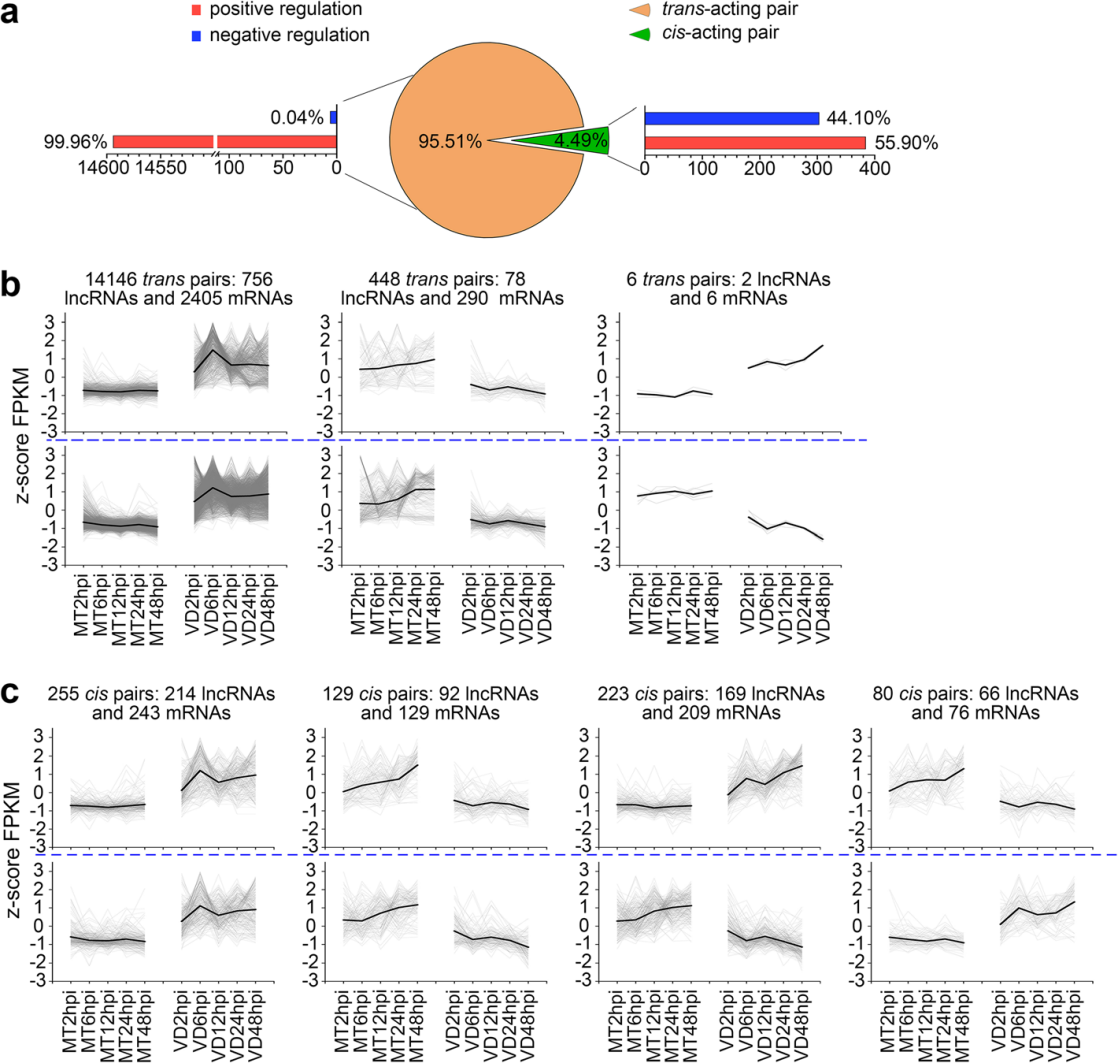
*Trans*-acting and *cis*-acting of differentially expressed lncRNAs. **a** Ratio of the *trans*-acting pair and *cis*-acting pair, and ratio of positive regulation and negative regulation. **b** The patterns of *trans*-acting delncRNAs regulating target mRNAs in cotton. Above the blue line are the lncRNAs expression patterns; Below the blue line represent mRNAs expression patterns. **c** The patterns of *cis*-acting delncRNAs regulation target mRNA in cotton. The meaning of the blue line is the same as on **c**

It has been reported that lncRNAs can modulate gene activity through *cis*-acting and/or *trans*-acting [44, 45]. To analyze the potential regulation patterns of the delncRNAs, we selected the differentially expressed protein-coding genes that composed the *cis*- or *trans*- pairs with delncRNAs. In this study, 687 *cis*-acting pairs of lncRNA-mRNA were identified, and, more importantly, a large number of *trans*-acting pairs (14,600 pairs) were found. Obviously, *trans*-acting pairs were absolutely predominant in identified lncRNA-mRNA target pairs, accounting for 95.51% (Fig. 3a), which indicated that *trans*-acting was the main way of *V. dahliae-*induced lncRNAs regulating the target mRNAs in cotton.

As shown in the Fig. 3b, *trans*-acting pairs could be divided into three patterns. Pattern 1 contained 14,146 lncRNA-mRNA target pairs with 756 up-regulated lncRNAs and 2405 up-regulated mRNAs; Pattern 2 included 448 lncRNA-mRNA target pairs with 78 down-regulated lncRNAs and 290 down-regulated mRNAs; Pattern 3 only had 6 lncRNA-mRNA target pairs with 2 up-regulated lncRNAs and 6 down-regulated mRNAs. Obviously, the lncRNA-mRNA target pairs belonging to pattern 1 and 2 were positive regulatory action pairs, while those belonging to pattern 3 were negative regulatory action pairs, accounting for 99.96 and 0.04%, respectively (Fig. 3a and b). These suggested that the influence of *trans*-acting lncRNAs on its predicted target protein-coding genes was dominated by positive regulation.

The *cis*-acting pairs were put in four patterns, based on regulatory model of lncRNAs to its target mRNAs (Fig. 3c). Pattern 1 included 255 lncRNA-mRNA target pairs consisting of 214 up-regulated lncRNAs and 243 up-regulated mRNAs; Pattern 2 contained 129 lncRNA-mRNA target pairs composing of 92 down-regulated lncRNAs and 129 down-regulated mRNAs; Pattern 3 exhibited 223 lncRNA-mRNA target pairs comprising of 169 up-regulated lncRNAs and 209 down-regulated mRNAs; Pattern 4 displayed 80 lncRNA-mRNA target pairs with 66 down-regulated lncRNAs and 76 up-regulated mRNAs. Obviously, positive regulatory action pairs were clustered into pattern 1 and pattern 2, and negative regulatory action pairs were divided into pattern 3 and pattern 4, accounting for 55.90 and 44.10%, respectively (Fig. 3a and c). Comparing to *trans*-acting lncRNAs, negative regulation played an equally important role as positive regulation in *cis*-acting lncRNAs regulating their target mRNAs.

### DelncRNAs participated in many disease resistance processes and exhibited a temporal specificity

**Fig. 4:**
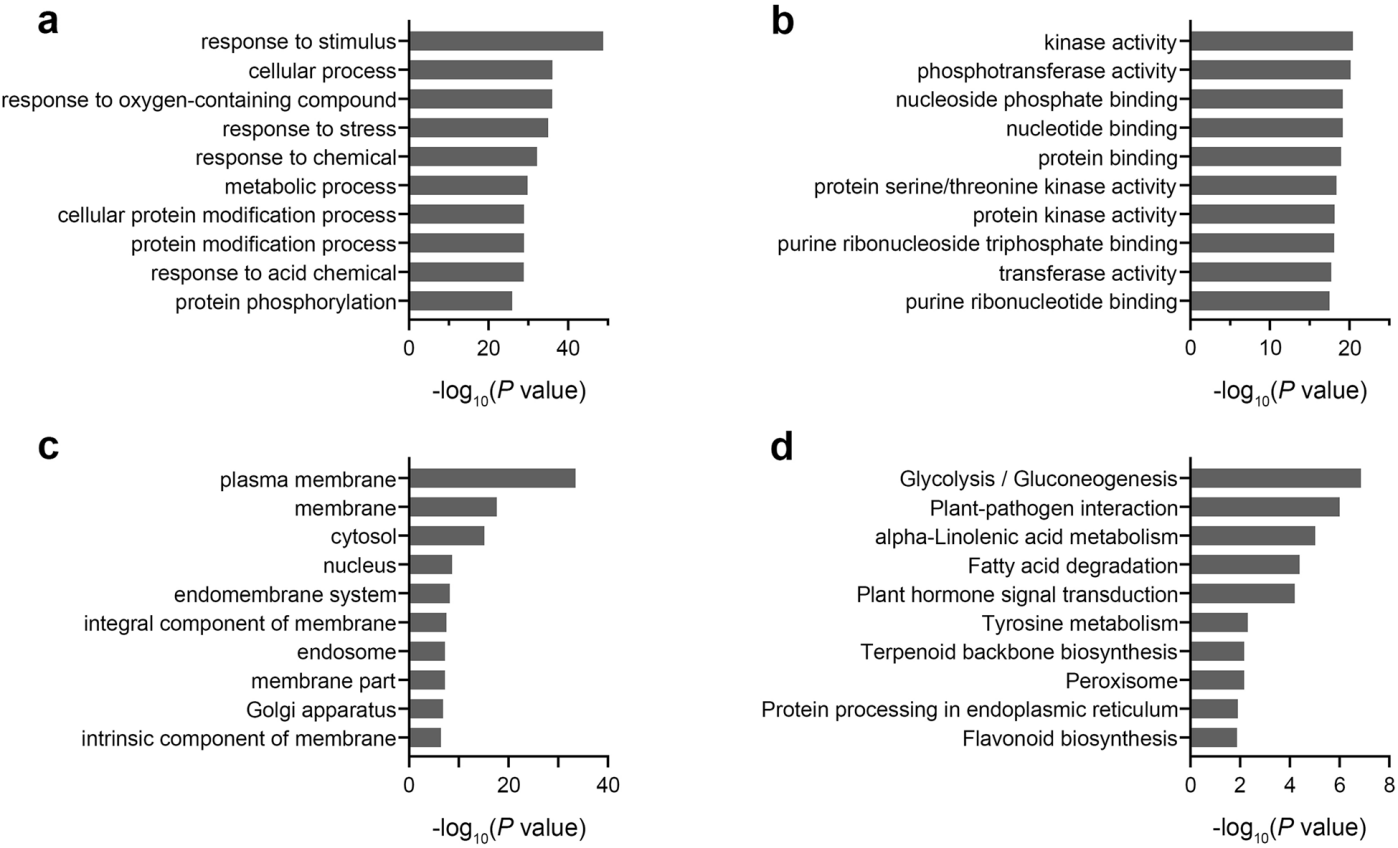
Disease resistance processes and function annotation of differentially expressed lncRNAs. The target mRNAs of delncRNAs were used for GO and KEGG enrichment analysis in order to predict their possible functions. GO terms are indicated by significant *P* values for each cluster. Here show the top 10 significantly enriched GO categories and pathways. **a** Biological process, **b** Molecular function, **c** Cellular component, **d** Pathways

In order to predict the potential function of delncRNAs, their target mRNAs were subjected to GO annotation and KEGG enrichment analysis. In the category of biological process, most of terms were related to stimulus response and protein modification (Fig. 4a). The top 10 terms of molecular function were related to kinase activity, protein binding and nucleotide binding (Fig. 4b). With regards to cellular components, biomembrane potentially played important role (Fig. 4c). In addition, the top 10 significant pathways were shown in Fig. 4d. It was interesting to note that glycolysis, plant-pathogen interaction, alpha-linolenic acid metabolism, fatty acid degradation, plant hormone signal transduction, terpenoid backbone biosynthesis, peroxisome and flavonoid biosynthesis were all associated with disease resistance, suggesting that these delncRNAs were implicated in responding to *V. dahliae* infection.

The GO enrichment analysis also showed that the function of unique delncRNAs varied with *V. dahliae* infection time (Fig. 2d). Concretely, it mainly related to stimulus response, kinase activity and phosphorylation at 2 hpi, stimulus response and hypoxia response at 6 hpi, stimulus response and single-organism process at 12 hpi, biomembrane at 24 hpi and cellular response to hypoxia at 48 hpi. These results indicated that lncRNAs could make a series of sequential responses rapidly to the infection of *V. dahliae* and further confirmed the dynamic characteristics of delncRNAs during cotton resistance to *V. dahliae*.

### Regulatory roles and characteristics of functional candidate lncRNAs in JA biosynthesis

**Fig. 5:**
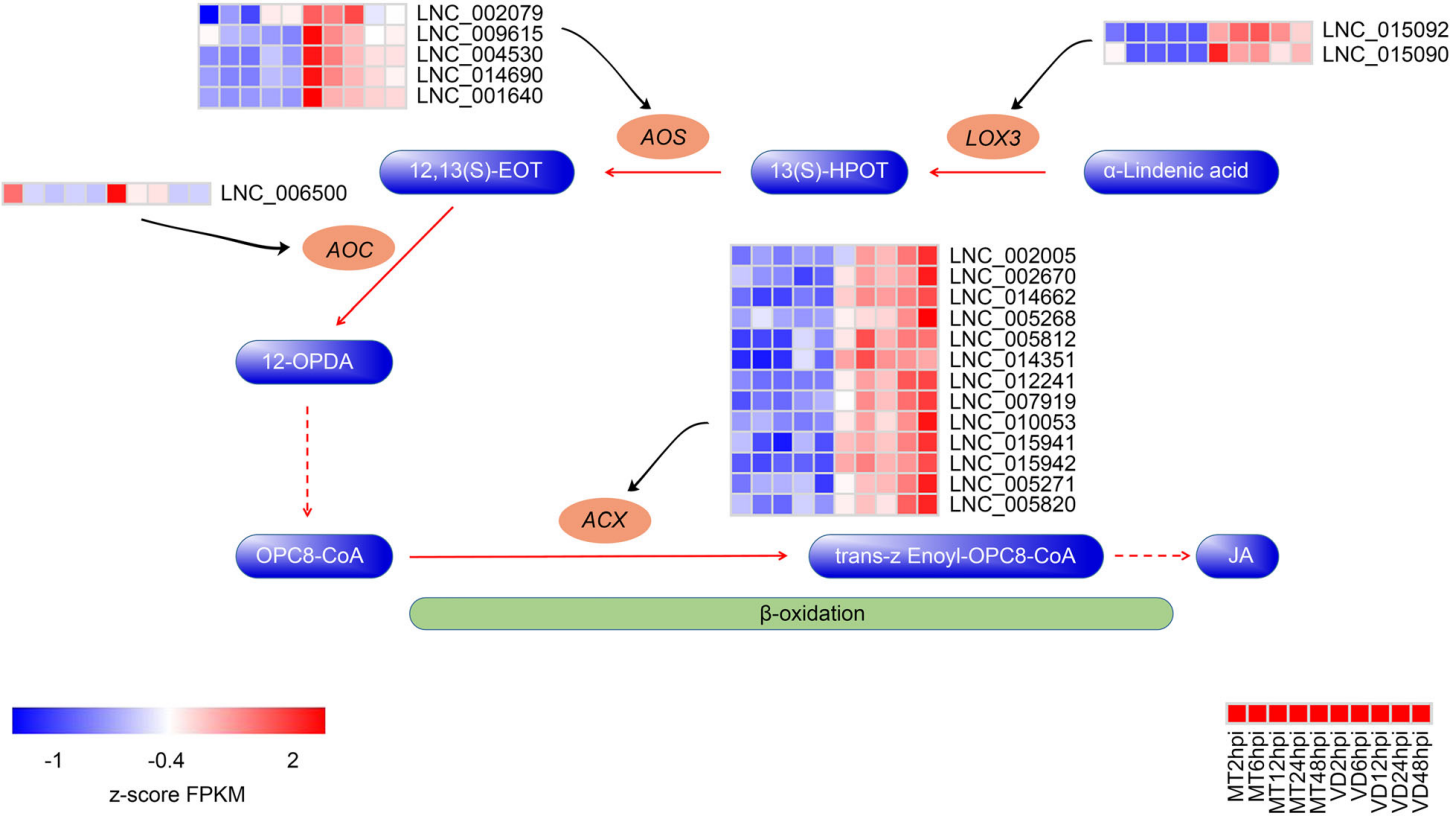
Differentially expressed lncRNAs involved in jasmonic acid (JA) biosynthesis pathway. The expression values of delncRNAs were normalized by z-score across all data sets. The red arrows represent one reaction step, and the dashed red arrows indicate a multi-step reaction. The black arrows indicate predicted regulatory effects of the lncRNAs on the target genes. 13(S)-HPOT represents 13S-hydroperoxy-6Z, 9Z, 11E-octadecatrienoic acid, an intermediate product of JA synthesis. 12, 13(S)-EOT represents (9Z, 15Z)-(13S)-12, 13-Epoxyoctadeca-9, 11, 15-trienoic acid. 12-OPDA represents (15Z)-12-Oxophyto-10, 15-dienoic acid

The result of KEGG enrichment analysis showed that alpha-linolenic acid metabolism was one of the significantly enriched pathways after infection with *V. dahliae*. Additionally, alpha-linolenic acid metabolism is associated with the biosynthesis of JA. Previous studies have showed that JA plays an important role in cotton resistance to *V. dahliae* [11]. In this study, 22 lncRNA-mRNA pairs (21 lncRNAs and 10 mRNAs) were found involving in JA biosynthesis pathway, and they were assigned to four gene families: lipoxygenase (LOX), allene oxide synthase (AOS), allene oxide cyclase (AOC) and acyl-CoA oxidase (ACX) (Additional file 3: Table S2). Specifically, two lncRNAs were involved in regulating two LOX family members, five lncRNAs had regulatory relationships with three target genes of AOS family members, one lncRNA could be a potential regulatory factor of two AOC family members, 13 lncRNAs participated in the regulation of three ACX family members. The expression profiles of 21 delncRNAs involved in JA biosynthesis pathways were analyzed based on the normalized FPKM values. The results showed that all the delncRNAs were up-regulated in the cotton roots attacked by *V. dahliae* (Fig. 5). The correlation analysis indicated that all of the 22 lncRNA-mRNA pairs were positively regulated (Additional file 3: Table S2). In the regulatory relationships between lncRNAs and their target genes, only five lncRNA-mRNA pairs were in a one-to-one regulatory relationship, and the remaining 17 lncRNA-mRNA pairs were not in a one-to-one regulatory relationship, such as *GhACX* (Gh_A09G2434) regulated by 11 lncRNAs (Additional file 4: Fig. S2). These results suggested that the regulatory roles of these 21 lncRNAs on their target mRNAs were complex.

### *GhlncLOX3* was up-regulated in response to *V. dahliae* invasion and potentially related to JA biosynthesis

LOX is an important enzyme involved in JA biosynthesis via alpha-linolenic acid metabolism pathway in plant. According to the analysis of lncRNA-mRNA pairs, we found that *GhLOX3* belonged to the target protein-coding gene of *trans*-acting lncRNA lnc_015092, designed as *GhlncLOX3*. Moreover, the expression of *GhlncLOX3* was up-regulated in all time points and peaked at 6 hpi for about 30 folds. Thus, we inferred that *GhlncLOX3* might be resistant to *V. dahliae* through regulating *GhLOX3* expression to influence JA synthesis.

**Fig. 6:**
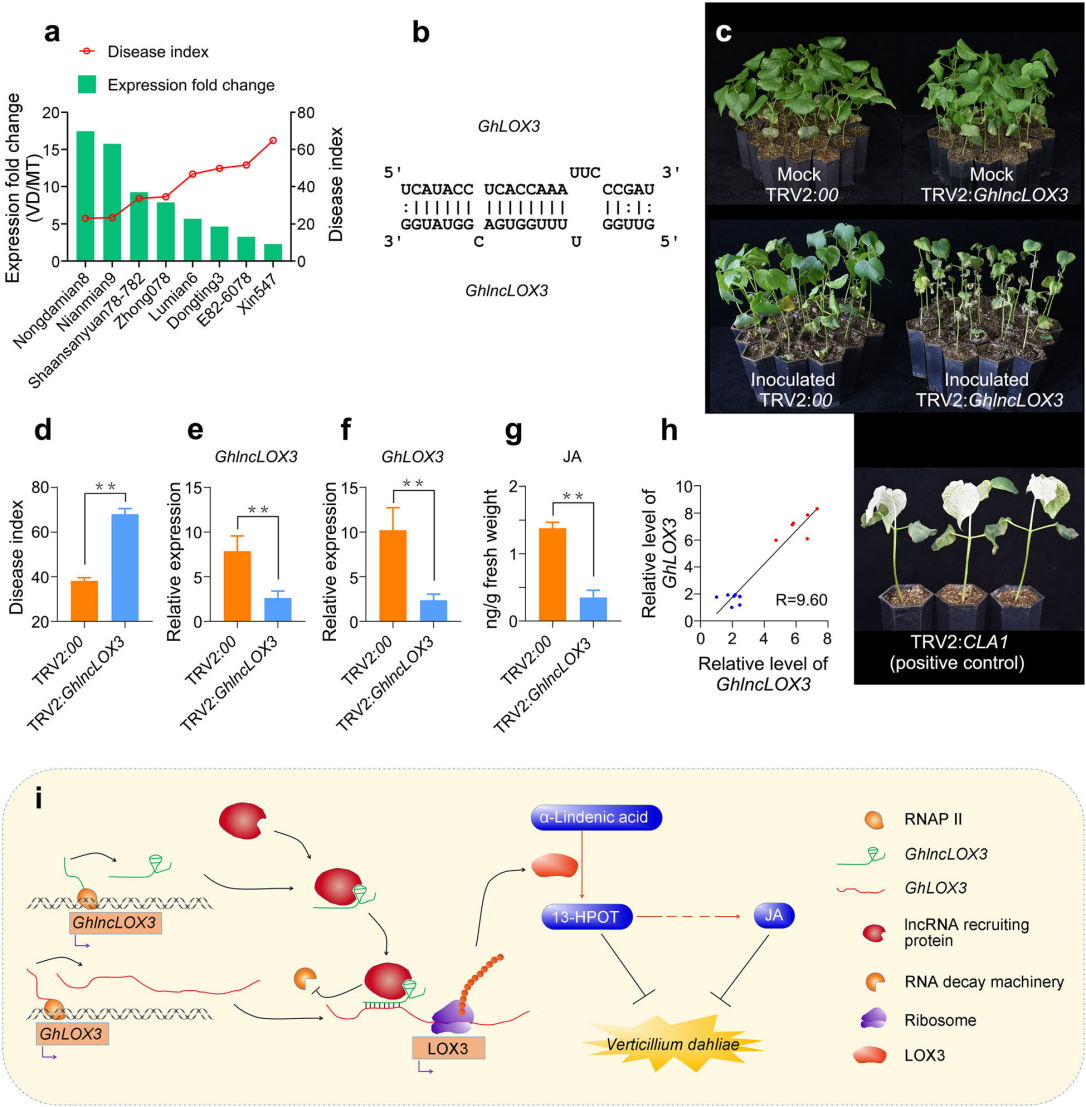
Functional identification of *GhlncLOX3* against *V. dahliae* using virus-induced gene silencing (VIGS). **a** *GhlncLOX3* gene expression fold change and disease index of infected plants in eight cultivars. ‘MT’ and ‘VD’ mean mock treatment and seedling roots inoculated with *V. dahliae*, respectively. **b** Predicted base-pairing interactions between *GhlncLOX3* and *GhLOX3*. **c** Phenotypes of seedlings with *GhlncLOX3* silencing post inoculation, showing the wilting and etiolated phenotype. *Cloroplastos alterados 1* (*CLA1*) was used as the positive gene. **d** Disease index of infected plants. **e** The qRT-PCR verification of *GhlncLOX3* silenced by VIGS. **f** Expression change level of *GhLOX3* after silencing *GhlncLOX3*. **g** Content change level of JA after silencing *GhlncLOX3*. **h** The correlation between *GhlncLOX3* (X-axis) and *GhLOX3* (Y-axis) expression levels in 14 samples was detected by Pearson correlation analysis. Red dots represent *GhlncLOX3*-silenced plants non-inoculated with *V. dahliae*. Blue dots represent *GhlncLOX3*-silenced plants inoculated with *V. dahliae*. **i** A schematic representation of speculative processes involved in *GhlncLOX3* raising the resistance of cotton to *Verticillium dahliae*. 13(S)-HPOT represents 13S-hydroperoxy-6Z, 9Z, 11E-octadecatrienoic acid, an intermediate product of JA synthesis. ** indicated significantly different (Student’s t-test, *P* < 0.01) in **d**, **e**, **f**, **g**

Further, quantitative real-time PCR experiments were carried out on eight cotton varieties with different resistance to *V. dahliae*. The results showed that *GhlncLOX3* was up-regulated in all varieties at 6 hpi. The fold up-regulation of *GhlncLOX3* was positively correlated with the disease resistance of cotton varieties (Fig. 6a). Moreover, the target gene *GhLOX3* has a similar expression trend with *GhlncLOX3* in all varieties (Additional file 5: Fig. S3). When *GhlncLOX3* was silenced via VIGS (Fig. 6e), the plants showed enhanced susceptibility to *V. dahliae* with more serious wilting and chlorosis of leaf comparing to CK (Fig. 6c). Additionally, the disease index (DI) of *GhlncLOX3*-silenced plants was significantly higher than that of controls at 25 dpi (day post inoculation) (Fig. 6d). Compared with the control, *GhlncLOX3*-silenced plants had lower expression level of *GhLOX3* and JA content (Fig. 6f and g). The correlation analysis indicated that the expression level of *GhLOX3* was positively correlated with that of *GhlncLOX3* (Fig. 6h). These results confirmed our hypothesis that *GhlncLOX3* affected JA synthesis by regulating the expression of *GhLOX3*.

IntaRNA program was executed to calculate potential binding sites between *GhlncLOX3* and *GhLOX3* [46]*.* As a result, a conceivable binding site with a free energy of − 17.54 k cal/mol was detected (Fig. 6b). LncRNA can exert its effects via direct mRNA binding and recruitment to some RNA binding proteins to regulate mRNA stability [47, 48]. Thus, we inferred that *GhlncLOX3* might block *GhLOX3* degradation via recruitment to some unknown RNA binding proteins. The result further suggested that *GhlncLOX3* regulated the disease resistance of cotton to *V. dahliae* via *GhlncLOX3*-*GhLOX3*-JA model. Based on the results of this study and previous reports, we proposed a schematic diagram for the role of *GhlncLOX3* in raising the resistance of cotton to *V. dahliae* (Fig. 6i).

## Discussion

LncRNA is a type of molecule with important functions in a wide range of biological processes across eukaryotes [44, 49]. It is a challenge to ascertain the biological function of lncRNAs due to the complex and diverse action mechanisms. Accumulating evidence suggests that lncRNAs can modulate some target genes via *cis*-acting and/or *trans*-acting [44, 45]. Nevertheless, most previous studies focused on *cis*-acting lncRNAs [1, 45, 50, 51]. For example, 92 disease resistance response *cis*-acting pairs of lncRNA-mRNA were identified in cotton [1]. In our research, the function of *trans*-acting lncRNA to resist *V. dahliae* in cotton was firstly established; and 14,600 *trans*-acting pairs of lncRNA-mRNA were identified as related to disease resistance (Fig. 3b). The number of *trans*-acting pair was 21 times as much as that of *cis*-acting pair, approximately. Therefore, we suggested that more attention should be paid to plant *trans*-action lncRNAs in the future. Moreover, we studied the regulation patterns of *cis*-acting lncRNAs and *trans*-action lncRNAs. The results unveiled that the regulation patterns of *cis*-acting lncRNAs and *trans*-acting lncRNAs on their target mRNAs were not exactly the same. *Trans*-acting lncRNAs were mainly positive regulation, while the negative regulation played the same important role as positive regulation in *cis*-acting lncRNAs regulating their target mRNAs. These results represent the first to characterize the important status and the regulation pattern of *trans*-acting lncRNAs involved in plant responses to infection by *V. dahliae*. In addition, our study is mutually complementary with the previous research of lncRNAs to resist *V. dahliae* in cotton, for resistant *G. hirsutum* is complementary to susceptible *G. hirsutum*, and *trans*-action lncRNA is complementary to *cis*-action lncRNA.

Verticillium wilt responsive lncRNAs had obvious dynamic characteristics (Fig. 2), which probably closely related to the infection process of *V. dahliae*. Recently, Zhang and Zhao monitored the infection processes of *V. dahliae* in root tissue of Arabidopsis and cotton, respectively [18, 52]. At 6 hpi, the conidia covered the root surface of both the Arabidopsis and cotton; By 24 hpi, massive conidia began to germinate and extended to form neogenetic hyphae; At 48 hpi, a mass of hyphae were formed in cotton root [18, 52]. In this research, the unique delncRNAs were enriched to stimulus response and hypoxia response at 6 hpi, which might be due to the fact that the local hypoxia of the root perimeter caused by spores covering; At 24 hpi, it manly related to biomembrane, which might be due to the fact that the extension of neonatal hyphae affected the membrane structure; At 48 hpi, it enriched to cellular response to hypoxia, which might be due to the fact that oxygen was snatched by a large number of hyphae, resulting in intracellular hypoxia in root tissue.

LncRNAs have a variety of complex mechanisms to regulate genes expression at multiple levels [1, 49, 53]. Recent works in plants have identified a large number of lncRNAs and greatly enhanced our knowledge on lncRNAs biology. however, the detailed functions and action mechanisms of lncRNAs are still in its infancy [38, 39, 49]. In this study, lncRNAs have been categorized into several GO and pathway terms based on annotation of target protein-coding genes (Fig. 4), which will help us to understand the potential functions of lncRNAs in response to pathogen infection.

Plants have to encounter various biotic and abiotic stresses throughout their development process. To deal with these adversities, plants have evolved constitutive and inducible defense systems [7, 11]. It is now clear that several endogenous signal molecules, such as salicylic acid (SA), ethylene (ET) and JA, are synthesized and can activate a series of complex defense signaling networks [11, 54]. Previous studies have showed that JA plays an important role in cotton resistance to *V. dahliae* [11]. Recently, 18 tea lncRNAs have been identified affecting JA biosynthesis through regulating the expression of JA-related genes [55]. Moreover, lncRNAs show low conservation among species [56]. In this study, 21 lncRNAs were found involving in JA biosynthesis pathway through complex regulatory networks.

LOX is a key enzyme of JA biosynthesis in plant and plays an important role in response to biotic stresses [57]. For example, it was indicated that *TomLoxD* might be resistant to *B. cinerea* through regulating JA biosynthesis to influence the expression of plant defense genes [58]; *CmLOX09* played a positive role in JA biosynthesis through the AOS pathway, and then increased resistance to *Podosphaera xanthii* [59]. In this study, transcriptomes data analyses showed a linkage between *GhlncLOX3* and *GhLOX3*, which prompted us to further investigate the function of *GhlncLOX3*. In order to figure out whether *GhlncLOX3* is involved in cotton resistance to Verticillium wilt and has a positive regulatory relationship with Gh*LOX3*, we tested the expression changes of *GhlncLOX3* and *GhLOX3* in eight different cotton varieties with *V. dahliae* infection. Moreover, *GhlncLOX3* was silenced using the VIGS technique, and we detected that *GhLOX3* and JA were significant down-regulated in *GhlncLOX3*-silenced plants. Thus, we preliminarily suggest that *GhlncLOX3*-*GhLOX3*-JA is an important network for cotton resistance to *V. dahliae*, although the further researches by RNA interference (RNAi) and over-expression need to be supplemented. In addition, a potential binding site between *GhlncLOX3* and *GhLOX3* was predicted (Fig. 6b). Recent studies have shown that lncRNAs can exert its effects via direct mRNA binding and recruitment to some RNA binding proteins to regulate mRNA stability [47, 48]. Therefore, we speculated that *GhlncLOX3* might enhance the stability of *GhLOX3* via nucleic acid binding and recruitment to some unknown RNA binding proteins. Although this conjecture needs further study to verify.

## Conclusions

In this study, using a high-throughput RNA-seq approach, 4277 delncRNAs are identified. And these delncRNAs show obvious dynamic characteristics in temporal specificity, chromosome distribution, induced expression profiles and biological function. Although previous studies pay more attention to *cis*-acting, this research reveals that *trans*-acting is the main way of *V. dahliae-*induced lncRNAs regulating the target mRNAs in cotton. Silencing of *GhlncLOX3* in cotton suppresses the accumulation of JA and reduces disease resistance, with down-regulation of *GhLOX3*. These results suggest that *GhlncLOX3*-*GhLOX3*-JA network plays an important role in cotton resistance to *V. dahliae* infection. Our results will extend the current view on lncRNA defence against *V. dahliae* infection and provide novel insights for genetic improvement of Verticillium wilt resistance in cotton.

## Methods

### Plant material and fungal pathogen

Resistant *G. hirsutum* cv. Nongda601 and Nongdamian8 were bred by our laboratory. Previously we also screened 419 accessions representing a core collection of cotton germplasm resources [60]. The disease index (DI) of 419 accessions and Nongda601 have been identified at 25 days post inoculation (dpi) by our laboratory using at least 30 seedlings, which have not been published. The seedlings of Nongda601 were used to build strand-specific cDNA libraries for transcriptome analysis. We selected 8 varieties (Nongdamian8, Nianmian9, Shaansanyuan78–782, Zhong078, Lumian6, Dongting3, E82–6078 and Xin547) with different resistance to *V. dahliae* from 419 core germplasm resources for further functional validation.

The cotton seedlings were grown in 50% Hoagland’s solution under greenhouse conditions of 25 °C for 4 weeks. Changed the nutrient solution, once every 4 days, in order to ensure the healthy growth of seedlings. A highly aggressive *V. dahliae* strain Linxi2–1 [61], was cultured on Potato Dextrose Agar (PDA) for 2 weeks from storage at 4 °C, and then sub-cultured into Czapek’s medium for 10 days on a shaker (130 rpm) at 25 °C. The conidial suspension was adjusted to a density of 10^7^ spores per milliliter with 50% Hoagland’s solution, as the final concentration for inoculation.

### RNA isolation and sequencing

When the fourth true leaf spread, the cotton seedlings of Nongda601 were inoculated with the spores of *V. dahliae*. According to our previous research about the infection process of *V. dahlia* [18], roots were harvested at 2, 6, 12, 24 and 48 hpi. As mock treatments, the plants treated with 50% Hoagland’s solution were also collected at the corresponding time points. High-quality total RNAs of all samples were extracted using the RNA prep Pure Plant Kit (TIANGEN Biotech, Beijing, China). rRNA was removed by Ribo-zero rRNA Removal Kit (Epicentre, USA). The strand-specific cDNA libraries were generated using the rRNA-depleted RNA by NEBNext Ultra Directional RNA Library Prep Kit for Illumina (NEB, USA) according to the manufacturer’s recommendations. Sequencing was performed on the Illumina Hiseq 4000 platform and 150 bp paired-end reads were generated.

### LncRNA identification

All sequence data were firstly processed by removing reads containing adapter, reads containing ploy-N and low-quality reads. The reference genome and the annotation files of *G. hirsutum* were downloaded from the CottonGen database (http://www.cottongen.org). Index of the reference genome was built using Bowtie2 v2.2.8 and paired-end clean reads were aligned to the reference genome using TopHat2 [62]. The mapped reads of each sample were assembled by Cufflinks [63]. According to the characteristics of lncRNA, we filtered the background noise based on Fragments Per Kilobase of exon per Million fragments mapped (FPKM), length, exon and coding potential (FPKM > 0.5; length > 200; exon ≥1; coding: NO).

### Expression analysis

Cufflinks provided computing program to calculate FPKM of both lncRNA and coding gene in each sample, and Cuffdiff provided statistical routines for determining differential expression data using a model based on the negative binomial distribution [63]. The differential expression transcripts were obtained by the following criteria: adjusted *P* value < 0.05 and at least two-fold FPKM change [1].

All pathway enrichment and GO terms of listed genes were annotated using KOBAS 3.0 (http://kobas.cbi.pku.edu.cn/) by comparing to the reference genome background (*P* < 0.01).

### Identification of *trans*-acting and *cis*-acting pairs

The correlation between lncRNA and mRNA was analyzed by Pearson correlation coefficient method to predict the target gene of lncRNA. The *trans*-acting pairs were obtained by the following criteria: absolute value of correlation > 0.95. *Cis*-acting is lncRNA acting on neighboring target genes. We set the threshold of *cis*-acting pairs to 100 kb, then lncRNA and mRNA with the same expression pattern were screened out by STME software [64].

### Cotton VIGS

As previously reported [1, 60], specific primers were designed to amplify *GhlncLOX3* fragment and cloned into the TRV2:*00* vector. Primer sequences are listed in Additional file 6: Table S3. Empty vector TRV2:*00* and containing candidate fragment vector TRV2:*GhlncLOX3* were, respectively, co-infiltrated with equal amount TRV1 via *Agrobacterium tumefaciens* GV3101 into cotton seedlings of Nongda601 when the cotyledons had spread. TRV2:*CLA1* (*CLOROPLASTOS ALTERADOS 1*) and TRV2:*00* were utilized as positive and negative control, respectively.

### VIGS plants inoculation

When the bleaching phenotype of positive controls appeared, we started to perform the inoculation with at least 30 plants for each treatment using *V. dahliae* strain Linxi2–1 with at least three biological replicates. At 6 hpi, leaves were harvested from several plants to assess *GhlncLOX3* silence degree and analyze changes in *GhLOX3* expression level and JA content. At 25 dpi, the phenotype was observed and the DI for plant populations was calculated according to previously described [61].

### Quantitative real-time (qRT) PCR analysis

Total plant RNA was extracted from cotton root or leaf using RNAprep Pure Plant Kit (TIANGEN Biotech, Beijing, China). The first stranded cDNA was synthesized from 0.5 μg RNA using the PrimeScript RT-PCR kit (TaKaRa, Dalian, China). Specific primers (Additional file 6: Table S3) were designed to implement the qRT-PCR on an ABI 7500 Real Time PCR system (Applied Biosystems, Waltham, Massachusetts, USA) with the SYBR *Premix Ex* Taq II system (TaKaRa, Dalian, China). The program of qRT-PCR was as follows: 95 °C for 2 min, followed by 40 cycles at 95 °C for 20 s, 55–60 °C for 20 s and 72 °C for 20 s. Gene expression levels were normalized to *GhUBQ14* (ubiquitin 14) expression [65].

## Supplementary Information

**Additional file 1: Table S1.** Summary of RNA -seq data.

**Additional file 2: Fig. S1.** The induced expression patterns of delncRNAs that were not included in Fig. 2c are shown here.

**Additional file 3: Table S2.** Correlation analysis of lncRNA-mRNA pairs related to JA biosynthesis pathway in cotton.

**Additional file 4: Fig. S2.** The regulatory relationships between lncRNAs and their target genes.

**Additional file 5: Fig. S3.** *GhLOX3* gene expression fold change and disease index of infected plants in 8 cultivars. ‘MT’ and ‘VD’ mean mock treatment and seedling roots inoculated with *V. dahliae*.

**Additional file 6: Table S3.** List of PCR primers used in this study.

## Data Availability

The data generated or analyzed during the current study are included in this published article and its supplemental data files and available from the corresponding author on reasonable request.

## Abbreviations

lncRNA: Long non-coding RNA
delncRNA: Differentially expressed lncRNA
GO: Gene Ontology
KEGG: Kyoto Encyclopedia of Genes and Genomes
miRNA: microRNA
*PR1*: *PATHOGENESIS-RELATED GENE1*
MED19a: Mediator subunits 19a
VIGS: Virus-induced gene silencing
hpi: Hours post inoculation
JA: Jasmonic acid
DI: Disease index
dpi: Day post inoculation
SA: Salicylic acid
ET: Ethylene
RNAi: RNA interference
PDA: Potato Dextrose Agar
*CLA1*: *CLOROPLASTOS ALTERADOS 1*
VD: *V. dahliae*-inoculated
MT: Mock-treated
