# Supplementary figures and images for: Dynamic characteristics and functional analysis provide new insights into long non-coding RNA responsive to Verticillium dahliae infection in Gossypium hirsutum
Source: BMC Plant Biol. 2021 Feb 1;21:68. doi: 10.1186/s12870-021-02835-8 (PMC7852192; doi:10.1186/s12870-021-02835-8)

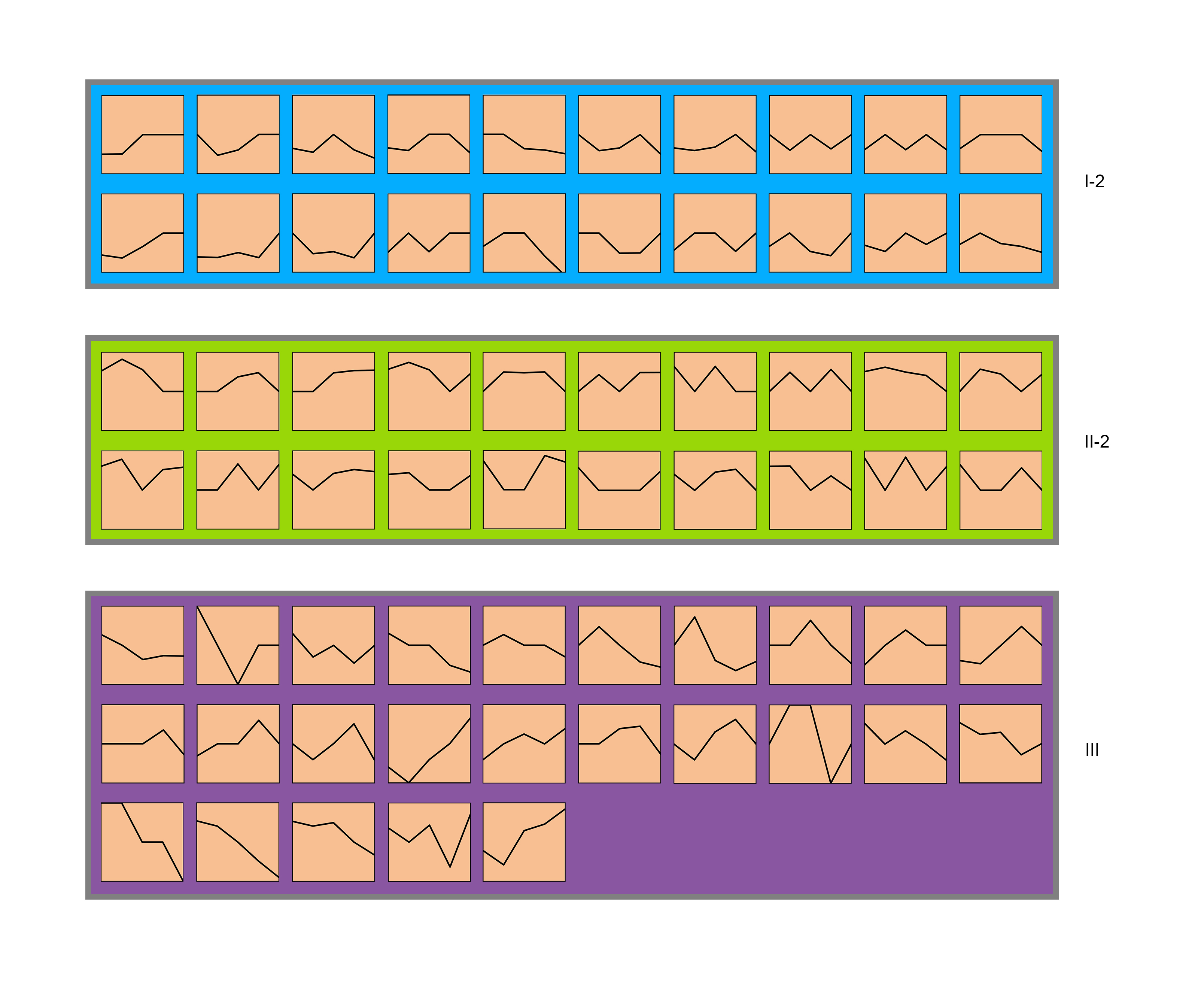

Supplement: Supplementary file 2 — Additional file 2: Fig. S1. The induced expression patterns of delncRNAs that were not included in Fig. 2c are shown here. [file 12870_2021_2835_MOESM2_ESM.jpg]

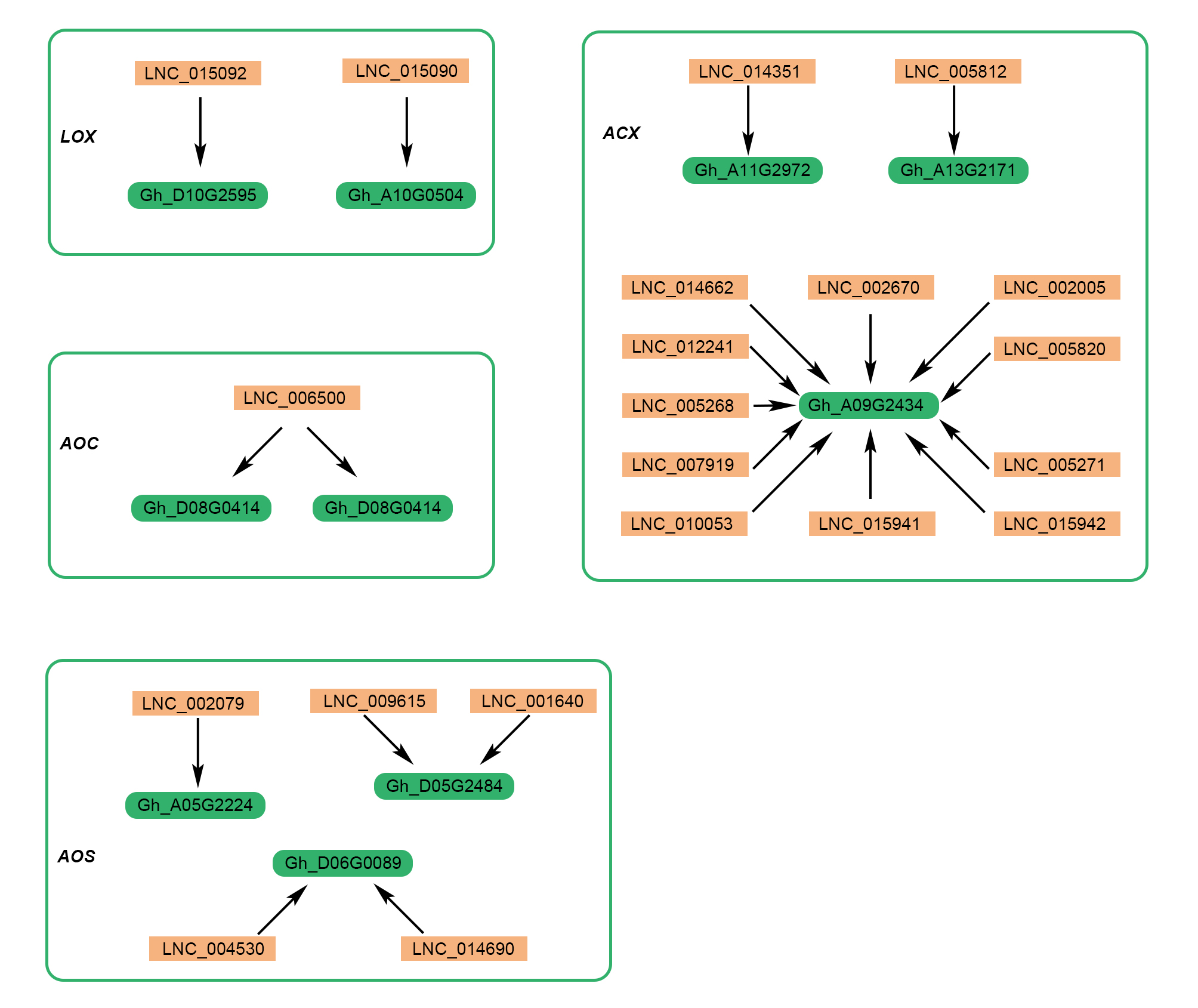

Supplement: Supplementary file 4 — Additional file 4: Fig. S2. The regulatory relationships between lncRNAs and their target genes. [file 12870_2021_2835_MOESM4_ESM.jpg]

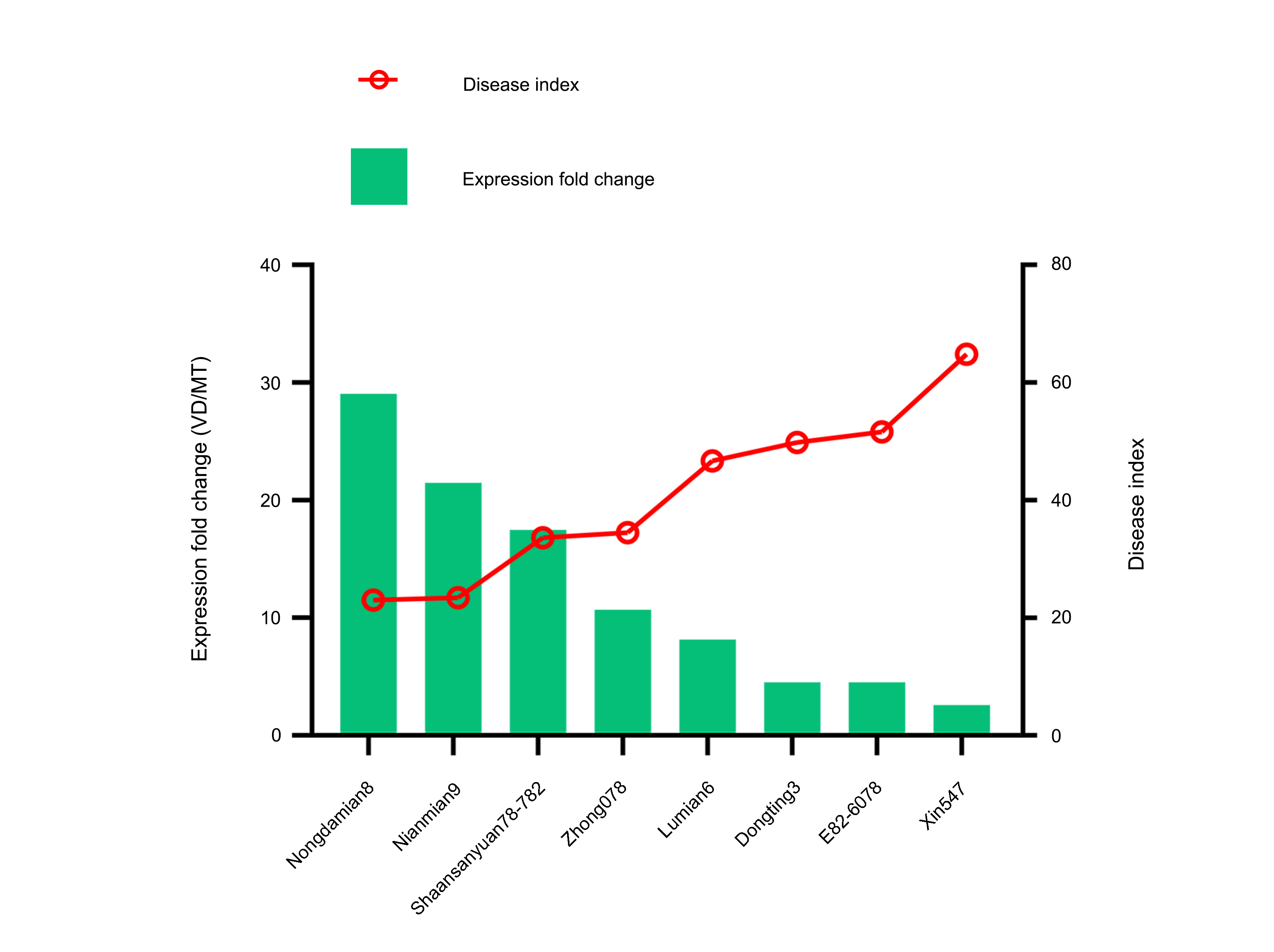

Supplement: Supplementary file 5 — Additional file 5: Fig. S3. GhLOX3 gene expression fold change and disease index of infected plants in 8 cultivars. ‘MT’ and ‘VD’ mean mock treatment and seedling roots inoculated with V. dahliae. [file 12870_2021_2835_MOESM5_ESM.jpg]
